# Safe duration of silicon catheter replacement in urological patients

**DOI:** 10.4314/gmj.v57i1.10

**Published:** 2023-01

**Authors:** Mawuenyo A Oyortey, Samuel A Essoun, Mahamudu A Ali, Mubarak Abdul-Rahman, James Welbeck, Jonathan C B Dakubo, James E Mensah

**Affiliations:** 1 Department of Surgery, School of Medicine, University of Health and Allied Sciences, Ho; 2 Department of Surgery, Korle Bu Teaching Hospital, Korle Bu, Accra; 3 Department of Pathology, University of Ghana Medical School, Korle Bu, Accra; 4 John Radcliffe Hospital, Oxford, England

**Keywords:** Urinary catheterisation, biofilms, encrustations, silicon catheter, catheter complications

## Abstract

**Objectives:**

This study compared the infection rates, degree of encrustation, symptoms, and complications in patients regarding the duration of urethral catheterisation (three weeks, six weeks, and eight weeks).

**Design:**

A cross-sectional study with stratified simple random sampling

**Setting:**

Urology Unit, Korle Bu Teaching Hospital

**Participants:**

One hundred and thirty-seven male patients with long-term urinary catheters

**Interventions:**

Participants were grouped into 3 weeks, 6 weeks, and 8 weeks duration of catheter replacements

**Primary outcomes measures:**

Symptoms due to the urinary catheters, urinalysis, urine and catheter tip cultures, sensitivity, and catheter encrustations were assessed.

**Results:**

Eighty-six patients had a primary diagnosis of benign prostatic hyperplasia (BPH), 35 had urethral strictures,13 had prostate cancer, two had BPH and urethral strictures, and one participant had bladder cancer. There was no difference in the symptoms the participants in the different groups experienced due to the urinary catheters (p > 0.05). The frequency of occurrence of complications (pyuria, p = 0.784; blocked catheter, p=0.097; urethral bleeding, p=0.148; epididymo-orchitis, p=0.769 and bladder spasms, p=1.000) showed no differences in the three groups. There was no statistical difference in the urinalysis for the three groups (p>0.05) and the degree of encrustations (3 weeks: 0.03 ± 0.06, 6 weeks: 0.11±0.27 and eight weeks: 0.12 ±0.27) with p=0.065.

**Conclusions:**

In this study, the duration of urinary catheterisation using silicone Foley's catheters did not influence the complication and symptom rates; hence silicon catheters can be placed in situ for up to 8 weeks before replacement instead of the traditional three-weekly change.

**Funding:**

Enterprise Computing Limited

## Introduction

Urinary catheterisation is common in 21.2% to 30% of hospitalised patients.^1^ In nursing homes in the United Kingdom, the overall prevalence was 9%, and the annual reported global estimate of urinary catheterisation is about 4 million patients.^2^ The insertion and use of a urinary catheter for more than 30 days is termed long-term indwelling urinary catheterisation.^3^ Currently, the overall prevalence of long-term urinary catheterisation is unknown, and the prevalence of urinary catheterisation specific to genitourinary diseases in Ghana has not been studied. Long-term catheterisation is beneficial in obstructive urological conditions. However, it is associated with numerous complications such as catheter blockage, urinary leakage, urinary tract infections, encrustations, bladder spasm, bladder cancer, urinary calculi, tissue damage, meatal erosion, urethral injury, haematuria, and catheter expulsion which affect the quality of life.^4^ Studies have suggested that latex catheters are more prone to developing these complications than silicon catheters, especially when the change duration is unknown.^5^

In certain parts of the world, the high patient-to-urologist ratio and inadequate healthcare financing delay the definitive urological interventions for patients giving rise to the increased burden of long-term catheterisation.^6^,^7^ As a result, catheter-related complications are higher. Prior to 2012, latex catheters were the most used Foley catheters in the Korle Bu Teaching hospital and were changed every 21 days (3 weeks). This practice was noted to be associated with an increased risk of severe catheter reactions, leading to long segment urethral strictures when a specific batch of latex Foley catheters was used between November 2011 and November 2012.^8^

This led to the introduction of silicone catheters, which are associated with fewer rates of complications and take longer to become blocked by encrustations.^9^,^10^ Frequent changing of these catheters increases the cost to the patient and predisposes them to the risk of complications. Without standardised recommendations for catheter replacement frequency, this study aimed at determining how long urethral and suprapubic catheters can be left in place without putting the patient at risk of catheter-related complications.

## Methods

### Study design and site

This cross-sectional study examined 137 patients with long-term indwelling urinary catheters for various urological indications changing their catheters in the Korle Bu Teaching Hospital's catheter room over one year. Using stratified simple random sampling, subjects were assigned to one of three categories (A=3 weekly changes, B=6 weekly changes, and C=8 weekly changes) with the 3-weekly group as the control after informed consent was obtained.

Patients with both urethral and suprapubic long-term silicon indwelling catheters, patients with obstructive uropathy secondary to bladder outlet obstruction who were having continuous bladder drainage as part of their management and required long-term catheterisation (suprapubic or urethral), and patients with neurogenic bladder who could not have intermittent catheterisation and were on long-term indwelling urethral or suprapubic catheter were eligible for the study.

### Data collection

A structured questionnaire was used to capture the patient's demographic characteristics, including the patient's diagnosis and the first catheterisation date. The patient's symptoms, including (urethral pain, suprapubic pain, flank pain, peri-catheter leakage, retention of urine, fever, penile or scrotal swellings) and any complications that occurred as a result of the catheterisation (urethral trauma, urethral bleeding, extravasation of urine, urethral discharge) were also recorded. The participant's weight and height were measured at their presentation to the catheter room, and BMI (body mass index) was calculated.

After the new catheter was inserted, a urine sample was collected into a sterile container, and the tip of the removed catheter was cut with a sterile blade on a sterile surface and placed in a sterile container for culture and sensitivity. Urine culture was done on Brilliant Green Agar (BGA) and Cysteine Lactose Electrolyte Deficient (CLED) Agar. The catheter tip smear was plated on blood, chocolate, and MacConkey agar smeared with thioglycollate broth and incubated for fastidious organisms overnight.

**Figure 1:**
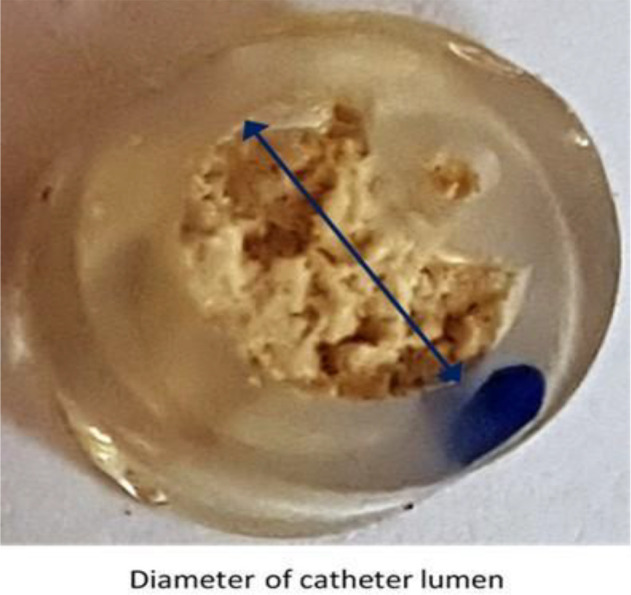
Cross section of the catheter showing encrustations.

The rest of the catheter tip was cut to a thickness of 0.3cm. Using a light microscope (ZEIS STEMI 1000) at a magnification of ×10, the catheter lumen diameter (Figure 1) and the width of the thickest part of the crust were measured using callipers (Castroviejo). The degree of catheter encrustation was determined as the ratio of the width of the thickest part of the crust to the catheter lumen diameter.

### Data Analysis

The data was entered into SPSS version 20. The relationship between patient symptoms, complications, urinalysis, urine culture, catheter tip culture, and catheter replacement duration were determined using the Chi-square and Fisher's exact tests. In addition, ANOVA was used to compare age, BMI, pH, and degree of encrustation among the study categories. Statistical significance was defined as a p-value of less than 0.05.

### Ethical Approval

Ethical approval was obtained from the College of Health Sciences Ethics and Protocol Review Committee of the University of Ghana with a Protocol Identification Number: CHS-Et/M.02- P 3.4/2015-2016.

## Results

The 135 patients ranged in age from 18 to 93 years old, with an average age of 67.29 ±12.81 years (Table 1). In addition, two patients had no idea of their ages or birth dates. Table 1 describes the mean ages for the three sub-groups. 27.0% of the patients had hypertension, 2.2% had diabetes mellitus, and 3.6% had both diabetes and hypertension; the majority (67.2%) had no co-morbidities.

**Table 1:** Mean ages by the duration of catheter replacement

	Duration	N	Mean ± Std. Deviation	P-VALUE
**AGE**	3 weeks	48	66.15± 14.22	0.644
	6 weeks	42	67.14 ± 10.55	
	8 weeks	45	68.64± 13.31	
	**Total**	**135**	**67.29 ± 12.81**	

The mean body mass index was 23.81 ± 6.35 kg/m2 (Table 2).

**Table 2:** Body mass index (BMI) by the duration of catheter replacement

	Duration	N	Mean ± Std. Deviation	P-VALUE
**BMI**	3 weeks	48	24.53 ± 7.07	0.297
	6 weeks	44	24.25 ± 5.49	
	8 weeks	45	22.61 ± 6.30	
	**Total**	**137**	**23.81 ± 6.35**	

The indications for the urinary catheterisations were benign prostatic hypertrophy (86 patients), urethral stricture (35 patients), prostate cancer (13 patients), combined urethral stricture and BPH (2 patients) and bladder carcinoma (1 patient). About 70.8% of the patients had urethral catheters, while 29.2% had suprapubic catheters.

The common catheter-related symptoms, as complained by the patients, were urethral pain (37 patients), peri-catheter leakage (31 patients), suprapubic pain (10 patients), urethral discharge (8 patients), flank pain and penoscrotal swelling, five patients each, and two patients had a fever. The distribution of symptoms is represented in Table 3.

**Table 3:** Patient symptoms by the duration of catheter replacement

	Duration of Catheter Replacement			
**Patient Symptoms**	**3 Weeks**	**6 Weeks**	**8 Weeks**	**p-value**
	n (%)	n (%)	n (%)	
**Urethral pain**				
**No**	35 (35.0)	31 (31.0)	34 (34.0)	0.205
**Yes**	13 (35.1)	13 (35.1)	11 (29.7)	
**Suprapubic pain**				
**No**	47 (37.0)	39 (30.7)	41 (32.3)	0.838
**Yes**	1 (10.0)	5 (50.0)	4 (40.0)	
**Flank pain**				
**No**	46 (34.8)	43 (32.6)	43 (32.6)	0.668
**Yes**	2 (40.0)	1 (20.0)	2 (40.0)
**Peri-catheter leakage**				
**No**	38 (35.8)	32 (30.2)	36 (34.0)	0.429
**Yes**	10 (32.3)	12 (38.7)	9 (29.0)	
**Urethral discharge**				
**No**	44 (34.1)	41 (31.8)	44 (34.1)	0.578
**Yes**	4 (50.0)	3 (37.5)	1 (12.5)	
**Retention of urine**				
**No**	48 (35.0)	44 (32.1)	45 (32.8)	
**Fever**				
**No**	48 (35.6)	43 (31.9)	44 (32.6)	0.420
**Yes**	0 (0.0)	1 (50.0)	1 (50.0)	
**Penile or Scrotal** **Swelling**				
**No**	47 (35.6)	43 (32.6)	42 (31.8)	0.528
**Yes**	1 (20.0)	1 (20.0)	3 (60.0)	

### Complications

The recorded complications in this study following urinary catheterisation were urethral bleeding, urethral discharge, pyuria (which was determined as >10 pus cells on urinalysis), blocked catheter, epididymo-orchitis and bladder spasms. Although there was no recorded urethral trauma, urethral stricture, stuck catheter, or bladder calculus from the three groups, most of the patients had py-uria (105), a blocked catheter (7) which were changed earlier, epididymo-orchitis (2) and bladder spasms (1).

### Urinalysis

Table 5 shows that the urine pH ranged between 5 and 9, with an overall mean of 7.07 ± 1.64. Microscopic haematuria was present in 119 patients, 63 patients had nitrites, 47 patients had crystals, leucocyte esterase was positive in 134 patients, 140 urine samples were positive for bacteria, and 2 urine samples showed the presence of yeast. Table 6 reports the urinalysis.

**Table 5:** Association between urine pH and duration of catheter replacement

	Duration	N	Mean ± Std. Deviation	p-value
**pH**	3 weeks	48	7.06 ± 1.45	0.707
	6 weeks	44	6.93 ± 2.00	
	8 weeks	45	7.22 ± 1.44	
	Total	137	7.07 ± 1.64	

**Table 6:** Association between urinalysis and duration of catheter replacement

	Duration of Catheter Replacement			
Urinalysis	3 weeks	6 weeks	8 weeks	p-value
**Blood/Haemoglobin**	n(%)	n(%)	n (%)	
+	4 (50.0)	3 (37.5)	1 (12.5)	
++	2 (33.3)	2 (33.3)	2 (33.3)	
+++	8 (38.1)	5 (23.8)	8 (38.1)	0.399
++++	27 (32.1)	32 (38.1)	25 (29.8)	
**Not detected**	7 (38.9)	2 (11.1)	9 (50.0)	
**Nitrite**				
**Negative**	26 (35.1)	22 (29.7)	26 (35.1)	
**Positive**	22 (34.9)	22 (34.9)	19 (30.2)	0.762
**Casts**				
**Cellular casts present**	0 (0.0)	1 (100.0)	0 (0.0)	
**Granular casts present**	2 (50.0)	0 (0.0)	2 (50.0)	0.784
**Not observed**	46 (34.8	43 (32.6)	43 (32.6)	
**Crystals**				
**Amorphous Phosphates**	5 (35.7)	2 (14.3)	7 (50.0)	0.519
**Amorphous urates**	1 (16.7)	1 (16.7)	4 (66.7)	
**Amorphous urates/ triple** **phosphate**	0 (0.0)	1 (50.0)	1 (50.0)	
**Calcium oxalate**	1 (50.0)	1 (50.0)	0 (0.0)	
**Not observed**	31 (34.4)	33 (36.7)	26 (28.9)	
**Triple phosphate**	3 (21.4)	5 (35.7)	6 (42.9)	
**Triple phosphate/ amorphous** **phosphate**	4 (66.7)	1 (16.7)	1 (16.7)	
**Uric acid**	3 (100.0)	0 (0.0)	0 (0.0)	
**Leucocyte Esterase**				
+	1 (100.0)	0 (0.0)	0 (0.0)	
++	1 (16.7)	4 (66.7)	1 (16.7)	0.210
+++	45 (35.4)	39 (30.7)	43 (33.9)	
++++	0 (0.0)	0 (0.0)	1 (100.0)	
**Not detected**	1 (50.0)	1 (50.0)	0 (0.0)	
**Bacteria**				
+	4 (66.7)	0 (0.0)	2 (33.3)	
++	6 (28.6)	6 (28.6)	9 (42.9)	
+++	35 (32.7)	38 (35.5)	34 (31.8)	0.376
				
**Not observed**	3 (100.0)	0 (0.0)	0 (0.0)	
**Yeast**				
**Absent**	46 (34.1)	44 (32.6)	45 (33.3)	0.329
**Present**	2 (100.0)	0 (0.0)	0 (0.0)	
**Urine Culture**				
**Negative**	37 (35.9)	30 (29.1)	36 (35.0)	0.405
**Positive**	11 (32.4)	14 (41.2)	9 (26.5)	

### Urine culture

**Figure 2:**
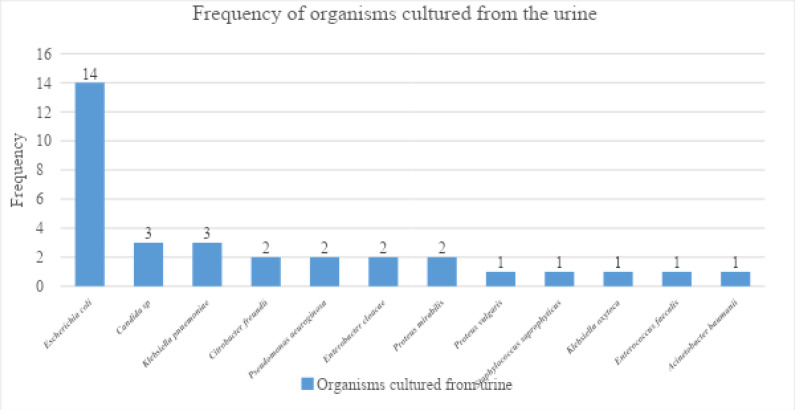
Distribution of organisms cultured from the urine.

There were 34 culture-positive urine specimens, with Escherichia coli being the most cultured organism (41%). The distribution of the cultured organism is shown in Figure 2.

### Catheter tip culture: Number of organisms

One hundred and twenty-two catheter tips cultured one organism on the Maki roll, while that for the intra-luminal culture was 112. Eight catheter tips (Maki roll) cultured 2 or more organisms, and 11 intraluminal cultures grew 2 or more organisms (Table 7).

**Table 7:** Number of organisms cultured by the duration of catheter replacement

Number of Organisms	Duration of Catheter Replacement			
MAKI ROLL	3 weeks	6 weeks	8 weeks	p-value
	Count (%)	Count (%)	Count (%)	
**0**	2 (28.6)	2 (28.6)	3 (42.9)	0.446
**1**	44 (36.1)	37 (30.3)	41 (33.6)	
**2 or more**	2 (25.0)	5 (62.5)	1 (12.5)	
INTRA-LUMINAL				
0	6 (42.9)	4 (28.6)	4 (28.6)	0.963
**1**	38 (33.9)	36 (32.1)	38 (33.9)	
**2 or more**	4 (36.4)	4 (36.4)	3 (27.3)	

### Organisms cultured

The most frequently cultured organisms for the external (maki roll) and intraluminal surfaces were similar. *Pseudomonas aeruginosa, Escherichia coli, Providencia rettgeri,* and *Klebsiella pneumonia* were predominant in the cultures, although *Pseudomonas aeruginosa* was frequently cultured on the external surface, while *Escherichia coli* was commonly found in the intra-luminal surface (Table 8).

**Table 8a:** Frequency of organisms cultured

Organisms cultured	N(%)
***Pseudomonas aeruginosa***	31(23.66)
***Escherichia coli***	19(14.50)
***Providencia rettgeri***	9(6.87)
***Klebsiellia pneumoniae***	8(6.11)
***Aeromonas hydrophilia***	8(6.11)
***Burkholderia capacia***	7(5.34)
***Enterococcus faecalis***	6(4.58)
***Proteus mirabilis***	4(3.05)
***Candida sp***	4(3.05)
***Enterobacter cloacae***	4(3.05)
***Klebsiella oxytoca***	3(2.29)
***Morganella morganii***	3(2.29)
***Providencia stuartii***	3(2.29)
***Pseudomonas fluorescens***	3(2.29)
***Raoultella ornitholytica***	3(2.29)
***Citrobacter koseri***	2(1.53)
***Citrobacter fruendii***	2(1.53)
***Pantoea sp***	1(0.76)
***Coagulase neg Staphylococcus***	1(0.76)
***Aeromonas salmonicidia***	1(0.76)
***Alkaligenes faecalis***	1(0.76)
***Proteus vulgaris***	1(0.76)
***Serratia odorifera***	1(0.76)
***Serratia marcescens***	1(0.76)
***Enterobacter aeurogenes***	1(0.76)
***Shawanella putrefaciens***	1(0.76)
***Vibrio alginolytica***	1(0.76)
***Achrobacter dentrifcans***	1(0.76)
***Ochrobactrum anthropi***	1(0.76)

**Table 8b:** Frequency of organisms at the catheter tip (Maki roll) cultured (intraluminal)

Organisms cultured	N(%)
***Escherichia coli***	28(20.89)
***Pseudomonas aeruginosa***	22(16.42)
***Providencia rettgeri***	11(8.21)
***Klebsiellia pneumoniae***	10(7.46)
***Aeromonas hydrophilia***	7(5.22)
***Morganella morganii***	7(5.22)
***Proteus vulgaris***	6(4.48)
***Enterococcus faecalis***	5(3.73)
***Citrobacter freundii***	4(2.99)
***Burkholderia capacia***	4(2.99)
***Klebsiella oxytoca***	3(2.24)
***Pantoea sp***	3(2.24)
***Providencia stuartii***	3(2.24)
***Candida sp***	3(2.24)
***Enerobacter cloacae***	3(2.24)
***Raoultella ornitholytica***	2(1.49)
***Pseudomonas fluorescens***	2(1.49)
***Proteus mirabilis***	2(1.49)
***Enterobacter aeurogenes***	1(0.75)
***Alkaligenes faecalis***	1(0.75)
***Citrobacter koseri***	1(0.75)
***Serratia odorifera***	1(0.75)
***Serratia marcesens***	1(0.75)
***Shawanella putrefaciens***	1(0.75)
***Ochrobactrum anthropi***	1(0.75)
***Staphlococcus aereus***	1(0.75)
***Achrobacter dentrificans***	1(0.75)

### Encrustations

Encrustation occurred on 35 catheter tips, and the mean degree of encrustation was 0.09+ 0.22 (Table 9).

**Table 9:** Degree of encrustation and duration of catheter replacement

	Duration	N	Mean ± Std. Deviation	p-value
**DEGREE OF ENCRUSTATION**	3 weeks	48	0.03± 0.06	0.065
	6 weeks	44	0.11 ±0.27	
	8 weeks	45	0.12± 0.27	
	Total	137	0.09 ± 0.22	

## Discussion

Urinary catheterisation is one of the most common procedures performed in the urology unit, but it is not without risks, which vary depending on the catheterisation technique, the length of time the catheter has been in place, the type of catheter material used, and the patient's co-morbidities, among other factors. This study evaluated the dangers of leaving the catheter in-situ for up to two months.

Some infection control programs have recognised that routinely changing urinary catheters when there is no indication (infection or blockage) increases the risk of urinary tract infections.^11^ Most practices followed the manufacturer's 30-day recommendation, contrary to best practices.^11^,^12^ Wilson, after reviewing the evidence pertaining to nursing actions for the prevalence of catheter-associated urinary tract infection (CAUTI) with short and long-term catheterisation, stated that the recommended time between catheter replacement depends on local policies and varies between one and three months.^13^

Studies have shown that patients on long-term urinary catheterisation because of conditions such as urethral strictures, prostatic diseases, and other urological conditions cover a widely diverse age range, as shown in this study (18-93 years).^14^,^15^ Depending on the cause, urethral strictures occur in people of varying ages, with inflammatory or infectious causes from sexually transmitted diseases occurring in the younger age group and traumatic causes occurring over varying age ranges.^16^ The common causes of urinary catheterisation in patients who presented with urine retention in Kumasi were benign prostatic hyperplasia, urethral stricture and prostate carcinoma.^17^ Similarly, in this study, 86 out of the 137 patients had BPH, followed by urethral stricture and carcinoma of the prostate.

In a study of urinary catheterisation in nursing homes in the United Kingdom, McNulty et al. discovered that 72% had urethral catheters, and the rest had suprapubic catheters.^2^ In comparison, in a study of patients with acute urinary retention and prolonged catheterisation in Nigeria, 47.4% had benign prostatic enlargement (BPE) and had urethral catheters, while 52.6% had urethral stricture disease with suprapubic catheters.^6^ As in the case of McNulty et al., this study had more patients with urethral (70.8%) than suprapubic (29.2%) catheters. Suprapubic catheterisation has been shown to be more comfortable than urethral catheters, and there is less risk of urethral injury and urethritis.^18^ However, no statistically significant difference existed between the catheterisation type and the catheter replacement duration. A p-value of 0.072 indicates that the type of catheterisation was not a confounding factor in the symptoms and complications experienced by the patients in the three groups.

The common problems or symptoms patients with long-term catheterisation experienced included urethral pain, suprapubic pain, peri-catheter leakage, urethral discharge, fever and penoscrotal swelling. In this study, 27% of the patients had urethral pain, 22.63% had peri-catheter leakage, 7.30% had suprapubic pain, 5.84% had urethral discharge, 3.65% had penoscrotal swelling, 1.46% had a fever. Several studies showed similar symptoms in patients with long-term urinary catheterisation.^14^,^19^ Similarly, in Nigeria, another study discovered that 69.4% of 62 patients with prolonged catheterisation had urethral/suprapubic pain, 32.3% had urethral bleeding, and 61% had peri-catheter leakage.^20^ Even though these symptoms were present, the study showed that the duration of catheter replacement did not influence the occurrence of these symptoms. Changing the catheter at 3 weeks or 8 weeks did not increase the incidence of these symptoms.

Other complications experienced by the participants in this study included urethral bleeding, urethral discharge, pyuria, blocked catheter, epididymo-orchitis, and bladder spasms. Stuck catheters, urethral strictures, and calculus were not seen in this study because these complications occur in patients who fail to change their catheters and keep them in for longer than necessary, as reported by Miason and Yenli, who reported two cases of vesical calculus after keeping their suprapubic and urethral catheters for 5 and 2 years, respectively.^21^ There was no iatrogenic injury, which may be because trained nurses changed the urethral catheters.

Bacteriuria is the most obvious evidence of urinary tract infection.^22^ Catheter-associated bacteriuria is defined by the presence of ≥10^5^ cfu/mL of ≥1 bacterial species in a single catheter urine specimen in patients without symptoms. For CAUTI, the patient should have symptoms with no other source of infection and ≥10^5^ cfu/mL of ≥1 bacterial species.^23^ Pyuria and leukocyte esterase indicate inflammation, which may occur even in the absence of infection (low specificity and positive predictive value), but its absence rules out an infection.^24^ Nitrites occur in urine when nitrate-reducing bacteria are present in the urine. Because not all organisms reduce nitrates in urine, the absence of nitrites does not rule out an infection. The duration of catheterisation has been determined to be the most important determinant of bacteriuria with a daily risk of 3-7%.^25^ Bacteriuria is reported as ‘few’ (+), ‘moderate’ (++), or ‘many’ (+++). In this study, bacteriuria was present in all the urine specimens analysed. The urine cultured bacteria in 34 (24.82%) and yeast in 2 urine samples. The difference between the groups was not statistically significant. Most urine cultures did not grow pathogenic bacteria, probably because some participants may have been on antibiotics. However, some showed mixed growth, probably from contamination, and it would have been ideal to repeat the urine cultures.

As expected, the catheter tip cultured more organisms than the urine samples. Studies have shown that although multiple bacteria can be detected on the catheter tip, only a small fraction would be determined in traditional microbiological cultures.^26^ The importance of organisms found in the biofilm that coats the catheter surface is that they are less susceptible to antibiotics because of the protective layer of the biofilm and the fact that organisms grow more slowly.^27^ The most common organisms for catheter-associated urinary tract infection and biofilm colonisation are *Escherichia coli, Proteus mirabilis* and *Pseudomonas aeruginosa*. Other studies included *Klebsiella pneumonia*.

The most common organisms cultured in the urine in this study were *Escherichia coli* (42%), followed by *Candida sp* and *Klebsiella pneumonia*. Risk factors for candida in the urine include diabetes mellitus and urinary drainage devices, all present in this study. The most commonly occurring organisms isolated from the catheter tip were *Pseudomonas aeruginosa, Escherichia coli, Providencia rettgeri* and *Klebsiella pneumonia*. Enteric bacteria are the most common cause of bacterial urinary tract infections. The groups were comparable in the number of organisms cultured from the catheter tip (p-values >0.05).

The mean pH for all the groups is less acidic than the normal urine pH (6.0). The presence of urea-producing bacteria in the urine, which make the urine less acidic than normal, also causes the build-up and deposition of salts, increasing crusts around the catheter and stone formation. The role of urease-producing organisms in the formation of bladder stones and crusts on catheters resulting in blockage, has been shown in several publications. 28,29,30

Finally, looking at the mean encrustation rates of the 3 groups, the 3 weeks group had a mean of 0.02 ± 0.59, the 6 weeks group 0.11 ± 0.27 and the 6 weeks group 0.12 ± 0.27. The mean degree of encrustation increased as the duration of the catheter stayed in place because the longer the catheter was in place, the more mineral content deposition on the catheter surface occurred, but this was not statistically significant.

## Conclusion

There was no difference in patient symptoms, complications, urinalysis, urine cultures, catheter tip and encrustation rates between the 3 weeks, 6 weeks, and 8 weeks groups of patients catheterised with the full silicone catheter. Therefore, we propose that catheters be left in place for patients undergoing long-term catheterisation using pure silicon catheters for up to 8 weeks before being changed.

## Figures and Tables

**Table 4:** Complications of catheterisation versus dura-tion of catheter replacement

	Duration of Catheter			

Complications of Catheterisation	3 weeks	6 weeks	8 weeks	p-value
**Urethral Bleeding**	n (%)	n (%)	n (%)	
**No**	44 (33.6)	42 (32.1)	45 (34.4)	0.148
**Yes**	4 (66.7)	2 (33.3)	0 (0.0)	
**Pyuria**				
**No**	11 (34.4)	9 (28.1)	12 (37.5)	0.784
**Yes**	37 (35.2)	35 (33.3)	33 (31.4)	
**Blocked Catheter**				
**No**	48 (36.9)	41 (31.5)	41 (31.5)	0.097
**Yes**	0 (0.0)	3 (42.9)	4 (57.1)	
**Epididymo Orchitis**				
**No**	47 (34.8)	43 (31.9)	45 (33.3)	0.769
**Yes**	1 (50.0)	1 (50.0)	0 (0.0)	
**Bladder Spasms**				
**No**	47 (34.6)	44 (32.4)	45 (33.1)	1.000
**Yes**	1 (100.0)	0 (0.0)	0 (0.0)	
